# Supernumerary Medial Rectus Muscle of the Orbit

**DOI:** 10.7759/cureus.21556

**Published:** 2022-01-24

**Authors:** Amer Alghamdi, Waleed Khayyat, Ramah Nazer, Abdullah Alowaid

**Affiliations:** 1 Family Medicine, King Abdulaziz Medical City, Ministry of National Guard Health Affairs, Jeddah, SAU; 2 Ophthalmology, King Khaled Eye Specialist Hospital, Riyadh, SAU; 3 Ophthalmology, Imam Abdulrahman Bin Faisal University, Dammam, SAU

**Keywords:** intraoperative care, oculomotor muscles, incidental findings, congenital abnormalities, strabismus

## Abstract

Supernumerary extraocular muscles (EOMs) are relatively rare in humans compared to other species. Therefore, few cases are reported on pediatric patients with strabismus.

We describe a case of an incidental intraoperative finding of a right eye accessory medial rectus (MR) muscle in a child with normal ocular motility. This supernumerary muscle was found inserted underneath the original MR muscle and was of a similar size. In this article, we discuss the anomaly and its clinical relevance.

## Introduction

Deep knowledge of the anatomy of the ocular muscles, compartments, vessels, nerves, and fascias is essential for the diagnosis and surgical treatment of orbital diseases. Voluntary movements of the eyeball are executed by six extrinsic ocular muscles. Restrictions of movements and functional disturbances of the eyeball are caused by the variation in the origin, position, and insertion of these muscles. Reported variations of extraocular muscles (EOMs) include congenital absence, supernumerary EOMs, and bifurcation of the muscle tendon. These are diagnosed as incidental findings on orbital imaging, post-mortem examinations, or during strabismus surgery [1]. Additionally, supernumerary EOMs are relatively rare in humans compared to other species [2], and they are noted mainly in cases of pediatric strabismus. We describe a unique presentation of a rare case of the unilateral anomalous medial rectus (MR) muscle and discuss its clinical relevance.

## Case presentation

An otherwise healthy nine-year-old girl, with no significant past medical, surgical, or trauma history, presented with an inward deviation of both eyes. The deviation started intermittently nine months before and progressed to be constant. Her best-corrected visual acuity was 20/30 and 20/20 in the right and left eye, respectively. Cyclo-refraction results were +3.50 sphere in the right eye and +3.00 sphere in the left eye with -1.00 cylinder in both eyes at 90 degrees. On initial examination, the patient was noted to have an esotropia (ET) of approximately 60 prism diopters (PD) at a distance with full ocular motility. No abnormal head or chin position was noted. MRI of the brain and orbits showed normal brain and parenchymal structures. An initial trial of full-correction glasses for six months was given. On subsequent visits, the deviation was found to be around 45 PD on full-correction glasses. 

**Figure 1:**
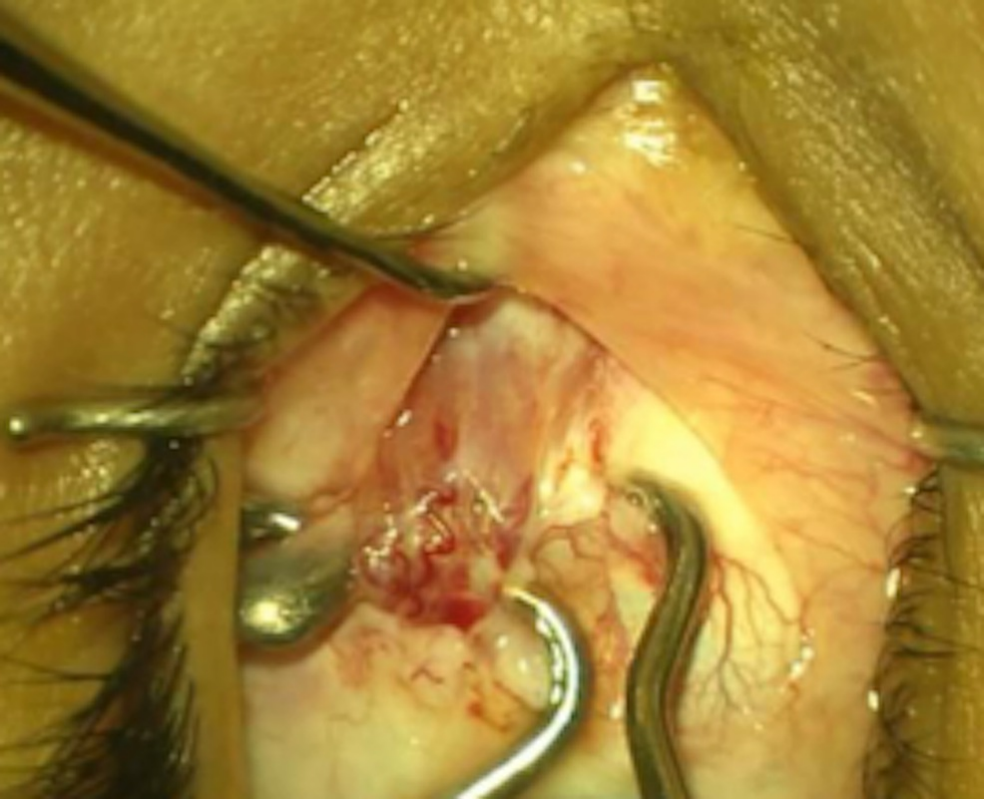
Fibromuscular band inserting behind the original medial rectus muscle.

**Figure 2:**
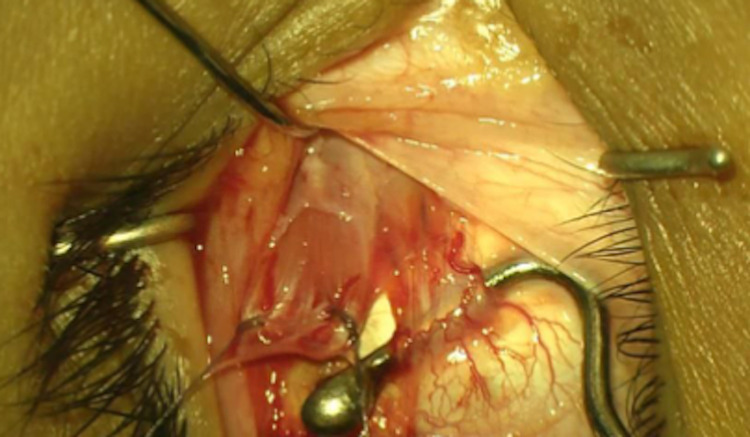
The original medial rectus muscle shown separately from the anomalous muscle.

We planned for bimedial rectus muscle recession (BMR), targeting 45 PD correction. Under general anesthesia, the surgery was started on the right eye with an inferonasal fornix-based incision. During hooking of the MR muscle, another fibromuscular band was incidentally found inserting behind the original MR muscle (Figure 1). The original MR was sutured, followed by the anomalous MR (Figure 2). A modified hang-back technique was used to recess the original MR muscle 5.5 mm away from the insertion. The same technique was used for the anomalous MR. Then, attention was shifted to the left eye with normal MR muscle anatomy and no anomalous muscles. A modified hang-back technique was used to recess the left eye MR muscle by 5 mm.

On the first post-operative visit, six weeks after the operation, the patient was found to be orthotropic in near and distance. In addition, she maintained full extraocular motility in both eyes.

## Discussion

Developmental anomalies of the EOM, apart from congenital fibrosis, are extremely rare in humans. Only a few case reports describe such anomalies [3]. The exact prevalence is unknown because of its rarity. The reason behind the presence of these accessory EOMs is yet unknown. One proposed reason is that it could be due to a disturbance in the mesodermal development [4]. Moreover, Whitnall in 1911 proposed another interesting hypothesis asserting that these fibromuscular bands of tissue may represent a homologous tissue to retractor bulbi muscles that are observed in some animals. This, then, pulls the eyes into the orbit for safety [5]. In our case, it was found to be an isolated, distinct muscle with features typical of an EOM, and no pathologic features were noted [4].

These anomalies, including accessory MR muscles, rarely affect the motility or the primary position of the globe [6]. However, some extreme abnormalities such as congenital EOM fibrosis or congenital absence of an EOM can disrupt ocular motility. In our case, the patient did not demonstrate any ocular motility abnormalities.

Despite accessory MR muscle being a rare entity, it could be a part of other associated ophthalmological or systemic diseases. For example, one case report stated that this entity might play a role in the pathogenesis of strabismus fixus convergence (a rare type of restricted strabismus) [7].

We did not perform histological confirmation of the lesion, as only recession of the anomalous muscle was performed. No abnormalities were identified on MRI of the brain and orbits. According to a previously published case series, small bands connecting two original EOM were observed in 12 subjects on MRI [8]. In our case, the accessory muscle was only coursing along the path of the original MR muscle with no apparent connection to another EOM. As these anomalous muscles rarely affect the overall motility of the corresponding muscle, it is recommended to continue with the intended surgical plan after an incidental finding of an accessory or two-bellied EOM [9].

## Conclusions

Accessory EOMs are infrequent incidental findings during strabismus surgery. This case of an accessory MR muscle provides an example of a rare anatomic anomaly. Awareness of such anomalies could assist ophthalmologists and radiologists in evaluating cases with unusual strabismus because this anomaly is manageable surgically.
